# Patient-reported outcomes and satisfaction after revisions of medial unicompartmental knee arthroplasties for unexplained pain vs aseptic loosening

**DOI:** 10.1007/s00167-023-07483-z

**Published:** 2023-07-27

**Authors:** Kristine Bollerup Arndt, Henrik Morville Schrøder, Anders Troelsen, Martin Lindberg-Larsen

**Affiliations:** 1 Department of Orthopaedic Surgery and Traumatology, Odense University Hospital, Odense, Denmark; 2 Department of Clinical Research, University of Southern Denmark, J. B. Winsløws Vej 4, 5000 Odense, Denmark; 3 Department of Regional Health Research, University of Southern Denmark, Odense, Denmark; 4 Department of Orthopaedic Surgery, Naestved Hospital. Naestved, Ringstedgade 61, 4700 Næstved, Denmark; 5 Departmentof Orthopaedic Surgery, Copenhagen University Hospital, Kettegård Alle 30, 2650 Hvidovre, Denmark

**Keywords:** Unicompartmental knee arthroplasty, Revision knee arthroplasty, Revision, Pain

## Abstract

**Purpose:**

Does patients revised for unexplained pain after mUKA present the same PROM and satisfaction scores 1–3 years after revision as patients revised for aseptic loosening?”.

**Methods:**

104 patients undergoing revision of mUKA's for the indications unexplained pain and aseptic loosening were included in the period January 1, 2018 to December 31, 2020.

from the Danish Knee Arthroplasty Register. 52 patients were revised for unexplained pain and 52 for aseptic loosening. Patient demographics did not differ between the two groups. PROMs [Oxford Knee Score (OKS), EQ-5D-5L, Forgotten Joint Score (FJS)] and questions about satisfaction with the surgery were sent to digitally secured mailboxes. Pearson’s Chi-square test and Wilcoxon Rank Sum test were used to test for statistical differences between groups.

**Results:**

The median OKS 1–3 years after revision was 26 (IQR 22) for unexplained pain vs 34 (IQR 12) for aseptic loosening, *p* = 0.033. The median EQ-5D-5L Index after revision was 0.7 (IQR 0.6) for unexplained vs 0.8 (IQR 0.1) for aseptic loosening, *p* = 0.014. The median FJS after revision was 48 (IQR 10) for unexplained pain vs 52 (IQR 14) for aseptic loosening, *p* = 0.1. The mean satisfaction with the surgery on a 0–100 scale (100 = not satisfied; 0 = very satisfied) was 55 (IQR 60) for unexplained pain vs 50 (IQR 67) for aseptic loosening, *p* = 0.087, and patients revised for unexplained pain were less likely to find their knee problem importantly improved (*p* = 0.032).

**Conclusion:**

Patients undergoing revision of mUKAs for unexplained pain presented poor postoperative PROM scores, and PROM scores were worse compared to those of patients revised for aseptic loosening. Patients revised for unexplained pain were less likely to find their knee problem importantly improved. This study support the evidence against revisions for unexplained pain.

**Level of evidence:**

Level III.

## Introduction

Medial unicompartmental knee arthroplasty (mUKA) is increasingly used, but 3–6 times higher 5 year revision rates of mUKA vs total knee arthroplasty (TKA) have been reported [28]. The surgeons usage of UKA vs TKA influences revisions rates of both with a decrease in UKA revisions when UKA usage is high [16, 17]. Recent studies have presented UKA revision rates approximating those of TKAs, probably due to improvements in UKA surgery and practice [2, 14]. The most common failure mechanisms for UKAs are aseptic loosening and progression of osteoarthritis, but a large proportion are revised because of unexplained pain. It has been reported that 36% of UKA revisions are performed for the indication aseptic loosening, 20% for progression of osteoarthritis, and 11% for unexplained pain [13, 26]. A large cohort study from the National Joint Registry of England and Wales (NJR) found that 23% of UKAs vs 9% of TKAs were revised for unexplained pain [1]. Revisions for unexplained pain are not recommended for neither UKAs nor TKAs [14, 29]. Pain revisions of UKAs are sparsely investigated, thus further evidence considering this topic is warranted in order to avoid unnecessary surgeries.

Studies investigating patient-reported outcome measures (PROMs) have found slightly better pain levels after UKA than TKA [2, 19, 20, 28]. However, more UKAs than TKAs are revised because of pain, probably because the threshold for revising a UKA is lower [8]. A nationwide investigation of revisions of UKAs based on data from the NJR demonstrated that 67% of UKAs were revised without any radiographic reason and the majority of these were performed for the indication unexplained pain [14]. In addition, many of the patients did not fulfil the radiographic criteria for primary UKA, and should not have undergone surgery in the first place [14]. It is uncertain if patients undergoing revision of UKAs because of unexplained pain benefit from the surgery, thus a PROM study using a control group of patients undergoing revisions for the more acknowledged indication aseptic loosening is warranted. Therefore the following research question was asked:

Does patients revised for unexplained pain after mUKA present the same PROM and satisfaction scores 1–3 years after revision as patients revised for aseptic loosening?”.

## Methods

This retrospective cross-sectional nationwide study was conducted in accordance with the COSMIN reporting guideline for PROM studies [7].

Data were collected on all revisions of mUKAs registered for the indications unexplained pain or aseptic loosening in the period January 1, 2018 to December 31, 2020 in the Danish Knee Arthroplasty Register (DKR). The DKR is a nationwide clinical database collecting data on primary and revision knee arthroplasties in Denmark since 1997 [24]. All orthopaedic departments, including private hospitals, report pre- and intraoperative data to the register. The completeness of the register was 97% for primary knee arthroplasties and 96% for revision knee arthroplasties in 2020 [5]. Demographic data on age, sex and BMI were retrieved from the DKR.

**Fig. 1:**
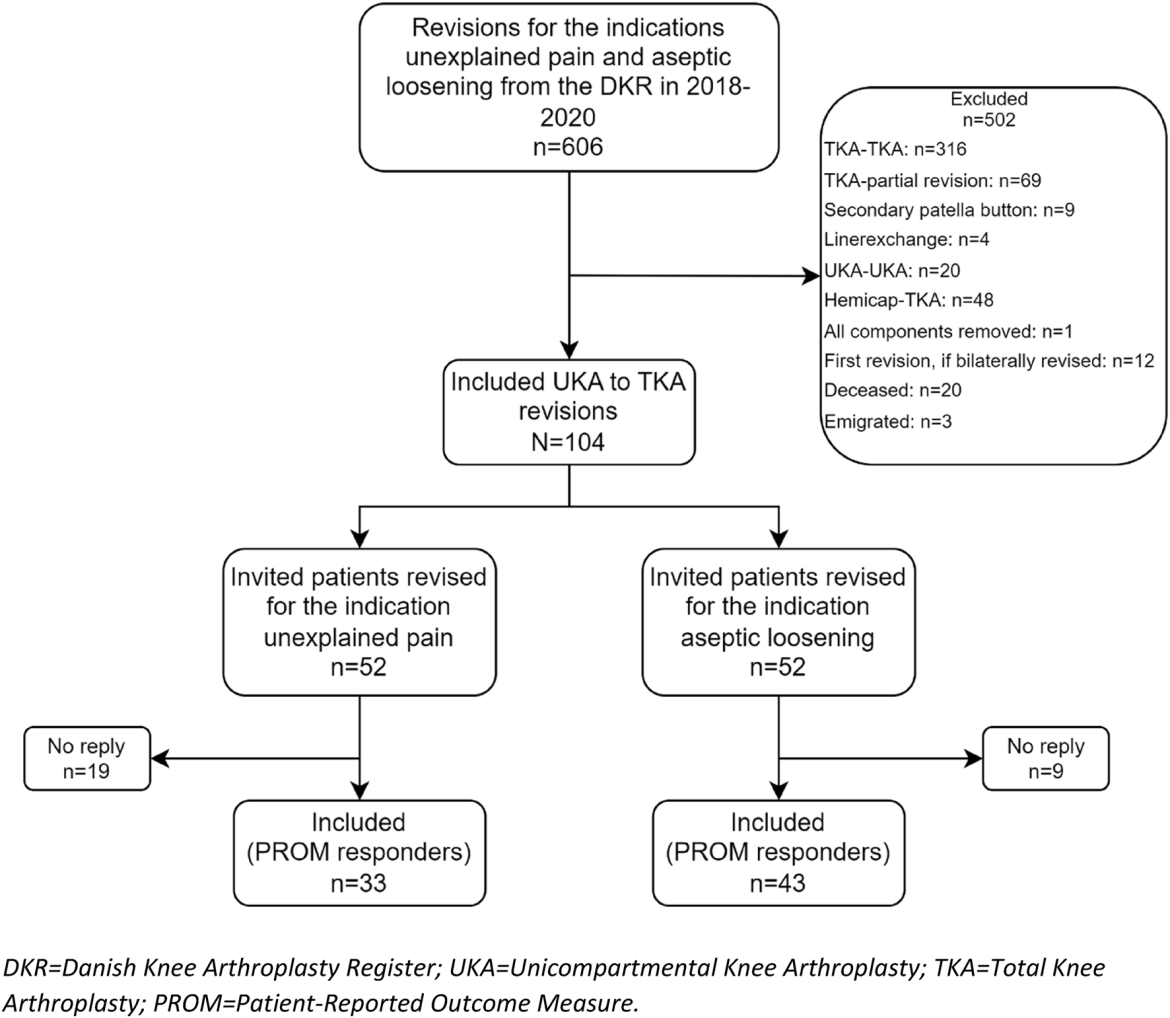
Flowchart of patients undergoing revision of medial unicompartmental knee arthroplasties for the indications unexplained pain or aseptic loosening included/excluded in this study

104 patients were included in the study, all of whom had a mUKA revised to a TKA (Fig. 1). Since all patients possible were included, a sample size calculation was not performed. 52 patients were revised for the indication unexplained pain and 52 for the indication aseptic loosening. Unexplained pain is an indication in the DKR that covers revisions of knee arthroplasties where no other obvious knee pathology are present. Aseptic loosening covers revisions where one of more of the components are assessed loose by the surgeon. The response rate was 73%. The demographic characteristics were similar for responders revised for unexplained pain vs aseptic loosening (Table 1). Types of prostheses are listed in Table 2. The study period of 2018 to 2020 was selected to ensure that time from revision to data collection would be within the time frame 1–3 years to diminish memory bias.

**Table 1:** Demographic characteristics of included patients undergoing revision of medial unicompartmental knee arthroplasties for the indications unexplained pain or aseptic loosening for responders and non-responders of patient-reported outcome measures

Characteristic	Responders		*p*-value	Non-responders		*p*-value
	Unexplained pain *n* = 33 (63%)	Aseptic loosening *n* = 43 (83%)		Unexplained pain *n* = 19 (37%)	Aseptic loosening *n* = 9 (17%)	
Age (Mean (range))	66 (39–80)	67 (43–85)	0.7	64 (44–84)	66 (52–83)	0.6
Female (%)	21 (64)	24 (56)	0.5	17 (89)	6 (67)	0.1
BMI (Median (IQR))	25 (10)	28 (9)	0.4	27 (17)	26 (12)	0.6
Follow-up (Years from revision to data collection) (Median (range))	2.4 (1.5–3.9)	3.3 (1.9–3.8)	0.02	3.4 (1.6–3.9)	3.3 (1.5–3.9)	0.6
Years from primary to revision surgery (Median (range))	5.5 (0.2–19)	5.7 (0.1–14.7)	0.7	4.6 (0.6–12.3)	4.1 (0.5–9.3)	0.4

*BMI* body mass index, *IQR* interquartile range. Statistical comparison of means has been performed with *t*-tests, comparisons of medians with Wilcoxon-rank sum tests, and proportions with Chi squared tests

**Table 2:** Types of medial unicompartmental knee arthroplasties presented by the indications unexplained pain or aseptic loosening for revisions

Company	Indication	
	Unexplained pain *n* = 52	Aseptic loosening *n* = 52
Oxford Partial knee	24	20
Endo-Model Sled Hemi (LINK)	1	–
NexGen Zuk	2	1
Sigma HP Partial Knee	1	–
Other	3	2
Unknown	21	29

## Outcomes

An email was sent to all included patients that linked to an electronic questionnaire in a secured digital mailbox. If the questionnaires were not answered within 2 weeks, two reminder emails were sent with a 2-week interval. Patients, who were not registered to the digital mailbox, received a paper version of the questionnaire by postal mail. Study data were collected and managed using REDCap electronic data capture tools hosted at Open Patient data Explorative Network (OPEN), Odense, Denmark [9, 10].

The questionnaires Oxford Knee Score (OKS), EQ-5D-5L, Forgotten Joint Score (FJS) and Copenhagen Knee ROM were used. The OKS score of 0–48 was calculated, with 48 being the best possible score [4, 23]. Calculation of the OKS followed recommendations from the developers [21]. The EQ Index was calculated from the United Kingdom value set, as a Danish value set does net jet exist [12, 27]. The FJS score was calculated following the instructions by the developers [3, 25]. The Copenhagen Knee ROM Scale was used to estimate the range of motion (ROM) of the knee [22].

The patients were asked about their level of pain and satisfaction after surgery (Table 4). Table 4 lists all questions on pain and satisfaction.

## Ethics

Permission from the Danish Data Protection Agency was achieved (Journal no. 19/14416). Accept to contact the patients in this study was achieved from the Head of Departments of all included departments performing the revisions. 

The authors had no conflicts of interest to declare.

## Statistics

Descriptive statistics were presented with means and standard deviations (SD) for normally distributed continuous variables and median and interquartile range (IQR) for non-normally distributed continuous variables. Distributions were inspected for normality via quartile–quartile-plots. Frequency counts and percentages were provided for categorical variables. Pearson’s Chi-square test was used to test for statistical differences between categorical measures. Wilcoxon Rank Sum test was used to test non-normally distributed continuous variables for statistical differences.

Missing data were as recommended by the developers for each of the PROMs [3, 6, 21].

Statistical significance was set at the 5% level. For all analyses, Stata Statistical Software were used: Release 17. College Station, TX: StataCorp LLC.

## Results

**Fig. 2:**
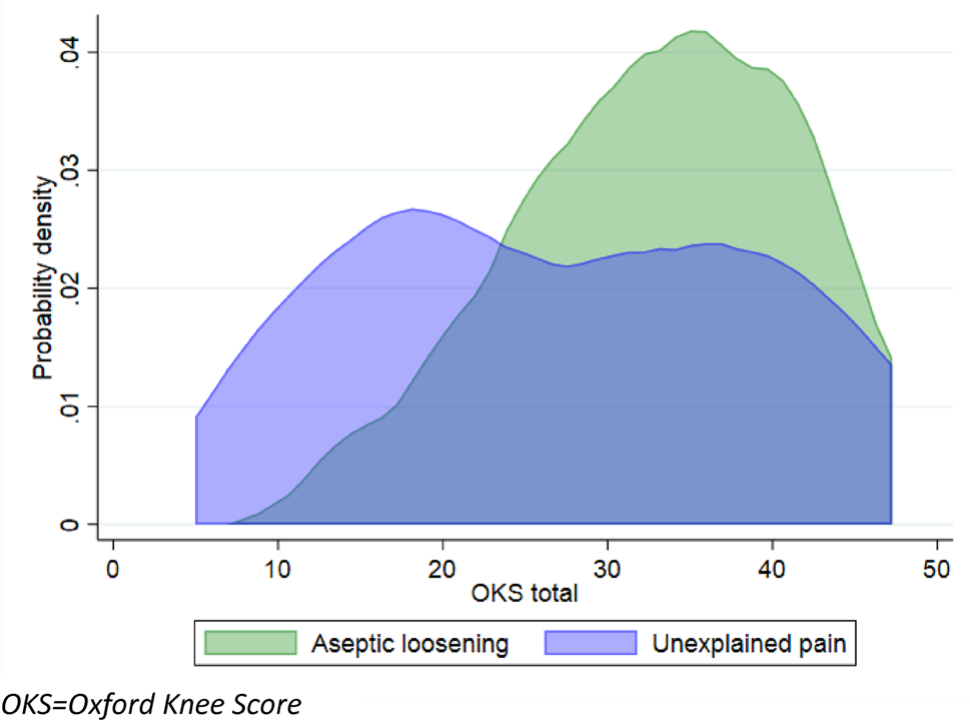
The distribution of OKS scores for patients undergoing revision of unicompartmental knee arthroplasties for the indications unexplained pain vs aseptic loosening presented as kernel curves. *OKS *Oxford Knee Score

**Fig. 3:**
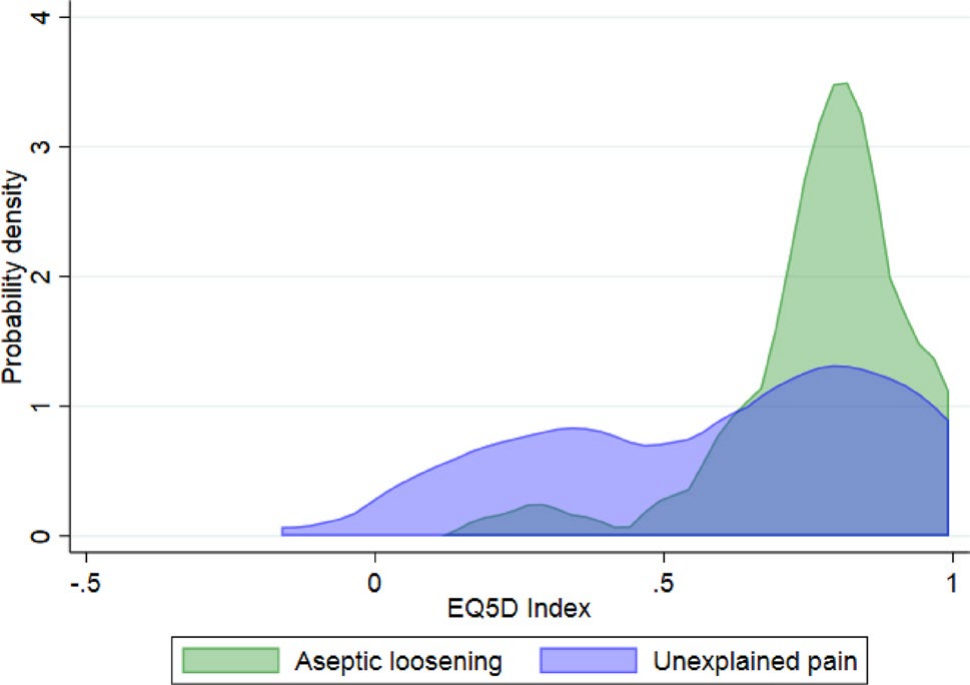
The distribution of EQ-5D Index scores for patients undergoing revisions of unicompartmental knee arthroplasties for the indications unexplained pain vs aseptic loosening presented as kernel curves

OKS, EQ-5D-5L Index, and EQ VAS scores were lower for patients revised for the indication unexplained pain than aseptic loosening (*p* = 0.033, *p* = 0.014, and *p* = 0.013) (Table 3 and Figs. 2, and 3).

**Table 3:** Patient-reported outcomes of patients undergoing revision of medial unicompartmental knee arthroplasties for the indications unexplained pain or aseptic loosening

PROM	Unexplained pain *n* = 33	Aseptic loosening *n* = 43	*p*-value
Oxford Knee Score	26 (IQR 22)	34 (IQR 12)	0.033
EQ-5D-5L Index	0.7 (IQR 0.6)	0.8 (IQR 0.1)	0.014
EQ VAS	50 (IQR 54)	75 (IQR 35)	0.013
FJS	48 (IQR 10)	52 (IQR 14)	0.1
Copenhagen Knee ROM			
Flexion	5 (IQR 2)	6 (IQR 2)	0.1
Flexion deficit (0–4)	12 (36%)	10 (23%)	n.s
Extension	3 (IQR 1)	4 (IQR 2)	n.s
Extension deficit (0–3)	17 (52%)	10 (23%)	n.s

*PROM* Patient-reported outcome measure, Chi-square test was used to compare proportions; Wilcoxon Rank Sum test was used to compare medians; IQR = Interquartile Range; EQ-5D-5L = EuroQol -5 Domain—5 Level—a value of 1 indicates the best quality of life and 0 indicate the worst; EQ VAS = EuroQol Visual Analogue Scale—100 = best health imaginable and 0 = worst health imaginable; Copenhagen Knee ROM = Copenhagen Knee Range of Motion

There were no significant differences in FJS or Copenhagen Knee ROM scores between indication groups, P = n.s. (Table 3).

**Fig. 4:**
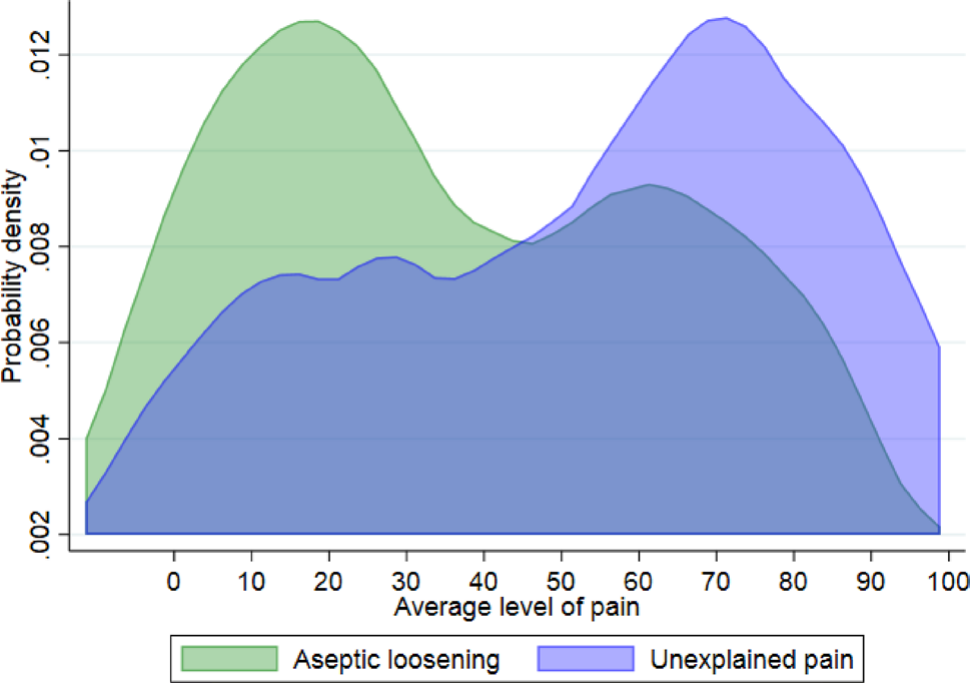
The distribution of pain scores (0 = no pain; 100 = worst pain imaginable) for patients undergoing revisions of unicompartmental knee arthroplasties for the indications unexplained pain vs aseptic loosening presented as kernel curves

The average score of pain was worse for unexplained pain than aseptic loosening (*p* = 0.043) (Table 4, Fig. 4).

**Table 4:** Questions on pain and satisfaction for patients undergoing revision of an unicompartmental knee arthroplasty for the indications unexplained pain vs aseptic loosening

Question	Unexplained pain *n* = 33	Aseptic loosening *n* = 43	*p*-value
Pain			
What was your average pain level the last month on a 0– 100 scale; 0 = no pain; 100 = worst pain imaginable^a^	54 (IQR 58)	33 (IQR 50)	0.043^b^
Satisfaction			
How satisfied are you with the result of the surgery on a 0–100 scale; 0 = very satisfied; 100 = not satisfied^a^	55 (IQR 60)	50 (IQR 67)	n.s
Improvement. How are your knee problems now compared to prior to the operation?			0.032
Importantly improved	14 (58%)	30 (83%)	
Not importantly improved	10 (42%)	6 (17%)	
Do you find your present situation acceptable considering your daily level of function?			n.s
Yes	15 (45%)	24 (62%)	
No	18 (55%)	15 (38%)	
The question was only asked to patients replying no to the above: Do you think the treatment has failed?			n.s
Yes	12 (71%)	9 (60%)	
No	5 (29%)	6 (40%)	
Would you go through the surgery again?			n.s
Yes	12 (36%)	21 (54%)	
Maybe	14 (43%)	9 (23%)	
No	7 (21%)	9 (23%)	

If no statistical test is mentioned for *p*-values, Chi-square test was used; *IQR* interquartile range

^a^Median (IQR)

^b^Wilcoxon Rank Sum test

The score for satisfaction with the surgery (100 = not satisfied; 0 = very satisfied) was low for both groups with a score of 55 (IQR 60) for unexplained pain vs 50 (IQR 67) for aseptic loosening, p = n.s. (Table 4). Patients revised for unexplained pain were less likely to find their knee problem importantly improved, *p* = 0.032 (Table 4).

## Discussion

The most important findings were, that patients revised for unexplained pain scored worse on OKS, EQ-5D-5L, and EQ VAS than patients revised for aseptic loosening. Further, the groups had similar low satisfaction rates with surgery, but a larger proportion of patients revised for unexplained pain did not consider the result of the surgery an important improvement.

OKS after TKA has been investigated previously. A study investigating Patient Acceptable Symptom State (PASS) values of OKS after primary TKAs found OKS scores of 30 (29–31) [11]. Though the study did not include revisions, the values are still relevant to our study. Our findings of OKS scores of 26 for patients revised for unexplained pain are below the acceptable state, whereas the scores for patients revised for aseptic loosening are acceptable. The minimal clinically important difference (MCID) after revision TKA are 4.9 [15]. Unfortunately, this study are not able to investigate MCID in the respective revision groups, but the difference within the two groups exceeds the MCID value. As Fig. 2 visualizes, the OKSs of patients revised for unexplained pain were biphasically distributed. This distribution suggests that some patients achieved an acceptable result whereas other patients did not. Patients who improved from the revisions might have had an underlying mechanical problem, which was solved from the revision. In general, patients revised for aseptic loosening performs acceptable after revision, but most of the patients revised for unexplained pain achieves unsatisfactory OKS scores after surgery.

A study of 578 patients undergoing aseptic revision of UKAs for all indications found a mean EQ-5D-5L Index score of 0.65 (SD 0.24) postoperatively [18], which is a considerably lower score than findings for both groups in the present study. The same study investigated VAS scores of satisfaction and pain, and found better scores than this study with a post-revision VAS for satisfaction (0–100 scale with 100 representing the best state) of 57 (SD 27) and VAS for pain of 61 (SD 23) [18]. Patients included in this study reported low levels of satisfaction after revision for both unexplained pain and aseptic loosening. However, patients revised for unexplained pain presented worse results than patients revised for aseptic loosening, and many of the patients did not improve considerably from the revisions. Revision of mUKAs for unexplained pain are not well regarded, and the results of this study support the reluctance against pain revisions. The outcomes of the pain revisions cannot be considered satisfactory, with only 58% of the patients revised for unexplained pain considering the surgery an important improvement and with the presentation of low PROM scores.

This is an important study providing data on a sparsely investigated area. The DKR is a nationwide register with a high level of completeness, and patients for this study were identified from this register. Acknowledged validated questionnaires were used for the study to gather the most reliable results possible.

There are limitations to this study. It was not possible to obtain a 100% response rate, and the missing responses might skew the results. However, a response rate of 73% was achieved, which was found adequate for a reliable analysis of data. PROMs prior to revision was not available for this study. It is a major limitation to the study, that the baseline status of the patients was unknown or if the groups differed initially on various PROMs. Furthermore, no information on the primary indications for the mUKAs were available, or if they were indicated according to good clinical practice.

## Conclusion

Patients undergoing revision of mUKAs for unexplained pain presented poor postoperative PROM scores, and PROM scores were worse compared to those of patients revised for aseptic loosening. Patients revised for unexplained pain were less likely to find their knee problem importantly improved. This study support the evidence against revisions for unexplained pain.

## References

[1] Baker PN, Petheram T, Avery PJ, Gregg PJ, Deehan DJ (2012). Revision for unexplained pain following unicompartmental and total knee replacement. J Bone Joint Surg Am.

[2] Beard DJ, Davies LJ, Cook JA, MacLennan G, Price A, Kent S (2019). The clinical and cost-effectiveness of total versus partial knee replacement in patients with medial compartment osteoarthritis (TOPKAT): 5-year outcomes of a randomised controlled trial. Lancet.

[3] Behrend H, Giesinger K, Giesinger JM, Kuster MS (2012). The "forgotten joint" as the ultimate goal in joint arthroplasty: validation of a new patient-reported outcome measure. J Arthroplasty.

[4] Dawson J, Fitzpatrick R, Murray D, Carr A (1998). Questionnaire on the perceptions of patients about total knee replacement. J Bone Joint Surg Br.

[5] DKR. The Danish Knee Arthroplasty Register, Annual Report 2021. 2021; 186. https://www.sundhed.dk/sundhedsfaglig/kvalitet/kliniske-kvalitetsdatabaser/planlagt-kirugi/knaealloplastikregister/. Accessed 16/8, 2022.

[6] EuroQol. EQ-5D User Guides. 2022; https://euroqol.org/publications/user-guides/. Accessed 16.06.2022, 2022.

[7] Gagnier JJ, Lai J, Mokkink LB, Terwee CB (2021). COSMIN reporting guideline for studies on measurement properties of patient-reported outcome measures. Qual Life Res.

[8] Goodfellow JW, O'Connor JJ, Murray DW (2010). A critique of revision rate as an outcome measure: re-interpretation of knee joint registry data. J Bone Joint Surg Br.

[9] Harris PA, Taylor R, Minor BL, Elliott V, Fernandez M, O'Neal L (2019). The REDCap consortium: Building an international community of software platform partners. J Biomed Inform.

[10] Harris PA, Taylor R, Thielke R, Payne J, Gonzalez N, Conde JG (2009). Research electronic data capture (REDCap)–a metadata-driven methodology and workflow process for providing translational research informatics support. J Biomed Inform.

[11] Ingelsrud LH, Roos EM, Terluin B, Gromov K, Husted H, Troelsen A (2018). Minimal important change values for the Oxford Knee score and the forgotten joint score at 1 year after total knee replacement. Acta Orthop.

[12] Jensen MB, Jensen CE, Gudex C, Pedersen KM, Sørensen SS, Ehlers LH (2021) Danish population health measured by the EQ-5D-5L. Scand J Public Health;10.1177/1403494821105806014034948211058060 10.1177/14034948211058060 PMC9969307 34847818

[13] Kamenaga T, Hiranaka T, Hida Y, Nakano N, Kuroda Y, Tsubosaka M (2022). Lateral osteoarthritis progression is associated with a postoperative residual tibiofemoral subluxation in Oxford UKA. Knee Surg Sports Traumatol Arthrosc.

[14] Kennedy JA, Palan J, Mellon SJ, Esler C, Dodd CAF, Pandit HG (2020). Most unicompartmental knee replacement revisions could be avoided: a radiographic evaluation of revised Oxford knees in the National Joint Registry. Knee Surg Sports Traumatol Arthrosc.

[15] Khow YZ, Liow MHL, Goh GS, Chen JY, Lo NN, Yeo SJ (2021). The Oxford knee score minimal clinically important difference for revision total knee arthroplasty. Knee.

[16] Klasan A, Parker DA, Lewis PL, Young SW (2022). Low percentage of surgeons meet the minimum recommended unicompartmental knee arthroplasty usage thresholds: analysis of 3037 Surgeons from Three National Joint Registries. Knee Surg Sports Traumatol Arthrosc.

[17] Klasan A, Tay ML, Frampton C, Young SW (2022). High usage of medial unicompartmental knee arthroplasty negatively influences total knee arthroplasty revision rate. Knee Surg Sports Traumatol Arthrosc.

[18] Leta TH, Lygre SH, Skredderstuen A, Hallan G, Gjertsen JE, Rokne B (2016). Outcomes of unicompartmental knee arthroplasty after aseptic revision to total knee arthroplasty: a comparative study of 768 TKAs and 578 UKAs revised to tkas from the norwegian arthroplasty register (1994–2011). J Bone Joint Surg Am.

[19] Liddle AD, Pandit H, Judge A, Murray DW (2015) Patient-reported outcomes after total and unicompartmental knee arthroplasty: a study of 14,076 matched patients from the National Joint Registry for England and Wales. Bone Joint J 97-b:793–801 10.1302/0301-620X.97B6.35155 26033059

[20] Mikkelsen M, Wilson HA, Gromov K, Price AJ, Troelsen A (2022). Comparing surgical strategies for end-stage anteromedial osteoarthritis : total versus unicompartmental knee arthroplasty. Bone Jt Open.

[21] Murray DW, Fitzpatrick R, Rogers K, Pandit H, Beard DJ, Carr AJ (2007). The use of the Oxford hip and knee scores. J Bone Joint Surg Br.

[22] Mørup-Petersen A, Holm PM, Holm CE, Klausen TW, Skou ST, Krogsgaard MR (2018). Knee osteoarthritis patients can provide useful estimates of passive knee range of motion: development and validation of the copenhagen knee ROM scale. J Arthroplasty.

[23] Mørup-Petersen A KM, Nielsen R, Paulsen A, Odgaard A. Translation and classical test theory validation of the Danish version of the Oxford Knee Score. [Abstract]. 2019; https://www.ortopaedi.dk/wp-content/uploads/2019/10/DOS-Abstract-bog-2019.pdf.

[24] Pedersen AB, Mehnert F, Odgaard A, Schrøder HM (2012). Existing data sources for clinical epidemiology: The Danish Knee Arthroplasty Register. Clin Epidemiol.

[25] Thomsen MG, Latifi R, Kallemose T, Barfod KW, Husted H, Troelsen A (2016). Good validity and reliability of the forgotten joint score in evaluating the outcome of total knee arthroplasty. Acta Orthop.

[26] van der List JP, Zuiderbaan HA, Pearle AD (2016). Why Do Medial Unicompartmental Knee Arthroplasties Fail Today?. J Arthroplasty.

[27] van Hout B, Janssen MF, Feng YS, Kohlmann T, Busschbach J, Golicki D (2012). Interim scoring for the EQ-5D-5L: mapping the EQ-5D-5L to EQ-5D-3L value sets. Value Health.

[28] Wilson HA, Middleton R, Abram SGF, Smith S, Alvand A, Jackson WF (2019). Patient relevant outcomes of unicompartmental versus total knee replacement: systematic review and meta-analysis. BMJ.

[29] Wylde V, Beswick A, Bruce J, Blom A, Howells N, Gooberman-Hill R (2018). Chronic pain after total knee arthroplasty. EFORT Open Rev.

